# Acyl coenzyme A binding protein (ACBP): An aging‐ and disease‐relevant “autophagy checkpoint”

**DOI:** 10.1111/acel.13910

**Published:** 2023-06-26

**Authors:** Léa Montégut, Mahmoud Abdellatif, Omar Motiño, Frank Madeo, Isabelle Martins, Victor Quesada, Carlos López‐Otín, Guido Kroemer

**Affiliations:** ^1^ Centre de Recherche des Cordeliers, Equipe labellisée par la Ligue Contre le Cancer, Inserm U1138 Université Paris Cité, Sorbonne Université Paris France; ^2^ Metabolomics and Cell Biology Platforms Gustave Roussy Institut Villejuif France; ^3^ Faculté de Médecine Université de Paris Saclay Paris France; ^4^ Department of Cardiology Medical University of Graz Graz Austria; ^5^ BioTechMed‐Graz Graz Austria; ^6^ Institute of Molecular Biosciences, NAWI Graz University of Graz Graz Austria; ^7^ Field of Excellence BioHealth University of Graz Graz Austria; ^8^ Departamento de Bioquímica y Biología Molecular, Instituto Universitario de Oncología del Principado de Asturias (IUOPA) Universidad de Oviedo Oviedo Spain; ^9^ Institut du Cancer Paris CARPEM, Department of Biology Hôpital Européen Georges Pompidou, AP‐HP Paris France

**Keywords:** aging, autophagy, diazepam‐binding inhibitor, endozepin, evolution, metabolism

## Abstract

Acyl coenzyme A binding protein (ACBP), also known as diazepam‐binding inhibitor (DBI), is a phylogenetically ancient protein present in some eubacteria and the entire eukaryotic radiation. In several eukaryotic phyla, ACBP/DBI transcends its intracellular function in fatty acid metabolism because it can be released into the extracellular space. This ACBP/DBI secretion usually occurs in response to nutrient scarcity through an autophagy‐dependent pathway. ACBP/DBI and its peptide fragments then act on a range of distinct receptors that diverge among phyla, namely metabotropic G protein‐coupled receptor in yeast (and likely in the mammalian central nervous system), a histidine receptor kinase in slime molds, and ionotropic gamma‐aminobutyric acid (GABA)_A_ receptors in mammals. Genetic or antibody‐mediated inhibition of ACBP/DBI orthologs interferes with nutrient stress‐induced adaptations such as sporulation or increased food intake in multiple species, as it enhances lifespan or healthspan in yeast, plant leaves, nematodes, and multiple mouse models. These lifespan and healthspan‐extending effects of ACBP/DBI suppression are coupled to the induction of autophagy. Altogether, it appears that neutralization of extracellular ACBP/DBI results in “autophagy checkpoint inhibition” to unleash the anti‐aging potential of autophagy. Of note, in humans, ACBP/DBI levels increase in various tissues, as well as in the plasma, in the context of aging, obesity, uncontrolled infection or cardiovascular, inflammatory, neurodegenerative, and malignant diseases.

## Graphical abstract

Acyl CoA binding protein (ACBP) encoded by diazepam‐binding inhibitor (DBI) is involved in homeostatic circuitries to adapt to changing food supply. In addition, ACBP/DBI participates in disease‐amplifying mechanisms relevant to aging, obesity, and a variety of major diseases.

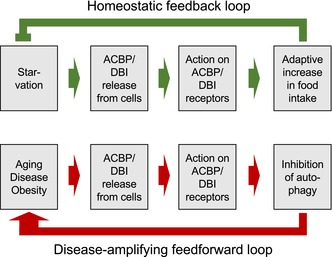

**Abbreviations**
ACBP: acyl CoA binding protein
AMPK: AMP‐activated protein kinase
CNS: central nervous system
CoA: coenzyme A
CVD: cardiovascular disease
DBI: diazepam‐ binding inhibitor
GABA: gamma‐amino butyric acid
GABRG2: γ2 chain of the GABA_A_ receptor
GPCR: GTP protein coupled receptor
KO: knockout
MPTP: 1‐methyl‐4‐phenyl‐1,2,3,6‐tetrahydropyridine
NASH: non‐alcoholic steatohepatitis
NDN: nonadecaneuropeptide
ODN: octadecaneuropeptide
TSP: translocator protein
TTN: triakontatetraneuropeptide

## 1 INTRODUCTION

In the human genome, acyl coenzyme A binding protein (ACBP) is encoded by *diazepam‐binding inhibitor* (DBI). This dual name, ACBP/DBI, reflects the scientific history of this protein, which has been amply studied for its capacity to bind to medium‐chain acyl coenzyme (CoA) esters that are often referred to as activated fatty acids. A large body of literature deals with the elucidation of the structure of ACBP/DBI alone or in complex with acyl CoA, as well as its implication in fatty acid metabolism (Du et al., 2016; Neess et al., 2015; Qiu & Zeng, 2020). Indeed, acyl CoA reportedly favors fatty acid oxidation by mitochondria by transporting activated fatty acid to this organelle (Duman et al., 2019; Knudsen et al., 1993). However, beyond its intracellular function as an acyl CoA‐binder, this highly conserved protein is also detected in the extracellular space including the plasma, where it has been discovered as an endogenous benzodiazepine (or “endozepine”) displacing a representative benzodiazepine (diazepam) from its receptors, explaining its denomination as DBI (Knudsen, 1991). The endozepine function of ACBP/DBI is shared by several of its peptide fragments that are referred to as “neuropeptides” and may have different lengths (e.g., triakontatetraneuropeptide, TTN, 34 amino acids corresponding to ACBP/DBI residues 17–50; octadecaneuropeptide, ODN, 18 amino acids corresponding to ACBP/DBI residues 33–50). Hence, another large body of literature has been treating the central nervous or behavioral effects of ACBP/DBI and the neuropeptides derived thereof (Alquier et al., 2021; Montegut et al., 2021).

**FIGURE 1:**
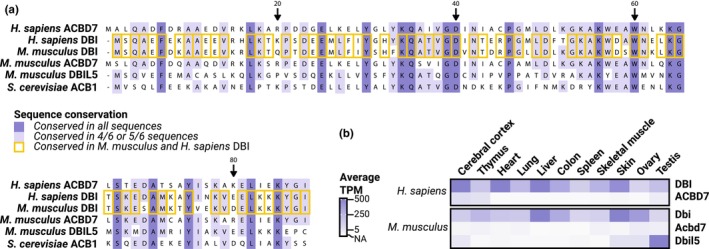
ACBP/DBI is the most abundantly expressed protein from a family of well‐conserved orthologs. Peptide sequence alignment reveals strong sequence similarities between the human and murine orthologs, and significant similarity with ACB1 from *Saccharomyces cerevisiae* (a). mRNA quantification (in average transcripts per millions, TPM) of the murine and human orthologs in different organs demonstrate that *DBI*/*Dbi* is the only ubiquitously expressed gene of the family (b) and is largely predominant in most organs of the periphery; TPM data were retrieved from consensus expression levels in www.proteinatlas.org for human genes (Uhlén et al., 2015), and average TPM by tissue from all available RNAseq samples from 8‐ 24‐week‐old wild type mice in the MGI database (Baldarelli et al., 2021), as consulted on April 12, 2023.

ACBP/DBI is a phylogenetically ancient gene/protein that exists already in some eubacteria and has been conserved throughout the eukaryotic radiation, meaning that its orthologs can be found in protists, fungi, plants, and animals (Faergeman et al., 2007; Thomas et al., 2021). It should be noted that the human genome encodes one close ACBP homologue, ACBD7. The genome of mice encodes a similar *Acbd7* gene plus *Dbil5*, which is also homologous to *Acbp* (Figure 1a). In contrast to ACBP/DBI which is expressed ubiquitously by most if not all cell types, *ACBD7*/*Acbd7* and *Dbi5* expression appears to be restricted to a few organs such as brain, testis, and ovary (Lanfray et al., 2016) (Figure 1b). Although multiple ACBP/DBI splice variants have been reported, only one single isoform (ACBP1) accounts for >90% of all *ACBP*/*DBI* transcripts in all human organs with the sole exception of the testis where it represents ~70% (Li et al., 2021). At this point, there is little or no information on the gonad‐ or brain‐specific functions of these mammalian ACBP/DBI homologues and isoforms apart from the anorexigenic effects of an ACBD7‐derived nonadecaneuropeptide (NDN) acting on the hypothalamus (Lanfray et al., 2016).

Fungal and animal cells secrete ACBP/DBI through an unconventional (Golgi‐independent) pathway that is autophagy dependent (Abrahamsen & Stenmark, 2010). Moreover, ACBP/DBI orthologs have been shown to regulate autophagy in fungi, plants, and animals (Bravo‐San Pedro, Sica, Martins, Anagnostopoulos, et al., 2019; Montegut, Joseph, Chen, Abdellatif, Ruckenstuhl, Motino, et al., 2023; Xiao et al., 2010; Xiao & Chye, 2010). Knockout of ACBP/DBI orthologs usually induces autophagy, suggesting that ACBP/DBI acts as an endogenous inhibitor (or “checkpoint”) of autophagy (Montegut, Joseph, Chen, Abdellatif, Ruckenstuhl, Martins, et al., 2023; Montegut, Joseph, Chen, Abdellatif, Ruckenstuhl, Motino, et al., 2023; Motino, Lambertucci, Anagnostopoulos, Li, Nah, et al., 2022). In connection with—or perhaps beyond—its autophagy‐regulatory role, ACBP/DBI has also been discovered to have a major impact on metabolism, hence influencing food intake, lipid anabolism and catabolism, body composition (Bravo‐San Pedro, Sica, Martins, Pol, et al., 2019), as well as aging and age‐related diseases (Fabrizio et al., 2010; Montegut, Joseph, Chen, Abdellatif, Ruckenstuhl, Motino, et al., 2023; Shamalnasab et al., 2017).

Motivated by these considerations, we provide an overview on the new roles of ACBP/DBI as a regulator of autophagy and metabolism. We will discuss the autophagy‐dependent, atypical secretion of ACBP/DBI in distinct eukaryotic phyla, evoke the evolutionary heterogeneity of its extracellular signaling functions and then summarize its (patho)physiological implication in primitive organisms, non‐human mammalian model organisms, as well as in humans.

## 2 AUTOPHAGY‐DEPENDENT SECRETION OF ACBP/DBI ACROSS PHYLOGENY

Across evolution, ACBP/DBI is a leaderless peptide, meaning that it lacks a signal sequence and cannot undergo conventional (Golgi‐dependent) secretion. ACBP/DBI orthologs undergo autophagy‐dependent secretion in several species ranging from unicellular fungi such as *Aspergillus oryzae* (ortholog: AoACBP) (Kawaguchi et al., 2016), *Pichia pastoris* (ortholog: Acb1) (Manjithaya et al., 2010), and *Saccharomyces cerevisiae* (ortholog: Acb1) (Bruns et al., 2011), to facultatively multicellular slime molds such as *Dictyostelium discoideum* (ortholog: AcbA, giving rise to the peptide fragment SDF‐2) (Duran et al., 2010), primary mouse astrocytes (Loomis et al., 2010), cardiomyocytes, hepatocytes, and skeleton muscle cells, as well as human peripheral blood mononuclear cells and carcinoma cell lines (Bravo‐San Pedro, Sica, Martins, Pol, et al., 2019). Reported triggers of ACBP/DBI release include different types of starvation (as demonstrated for all species) such as nitrogen starvation for fungal species, culture of mouse and human cells in nutrient‐free media, or fasting of mice for 24 h (Bravo‐San Pedro, Sica, Martins, Pol, et al., 2019). Of note, this ACBP/DBI release is not inhibited by brefeldin A, an inhibit of the classical (Golgi‐dependent) secretion pathway (Bravo‐San Pedro, Sica, Martins, Pol, et al., 2019; Loomis et al., 2010).

In *D*. *discoideum*, a cascade of hormones involving first the steroid hormone SDF‐3 (which can be mimicked by the glucocorticoid hydrocortisone) and subsequently gamma‐aminobutyric acid (GABA) induces the release of AcbA (Anjard & Loomis, 2006). Direct pharmacological stimulation of autophagy (without starvation) with rapamycin is sufficient to trigger ACBP/DBI release from mammalian cells (Bravo‐San Pedro, Sica, Martins, Pol, et al., 2019; Loomis et al., 2010). Moreover, glucocorticoids have been shown to stimulate the secretion of ACBP by primary mouse astrocytes (Loomis et al., 2010). In the plant *Arabidopsis thaliana*, one among 6 ACBP homologs, ACBP3, is present in the extracellular space (Leung et al., 2006). ACBP3 can be induced by pathogens and is subjected to circadian regulation (Zheng et al., 2012). It appears plausible, yet remains to be confirmed, that ACBP3 is secreted through an autophagy‐dependent process, which is known to be subjected to marked circadian fluctuations in plants (Chen et al., 2022; Yang, Zhu, et al., 2022).

The exact mechanism through which autophagy contributes to the release of ACBP/DBI in different species is largely elusive, requiring further molecular exploration. In mammalian cells, knockout of autophagy‐relevant genes (such as Atg5/ATG5 and Atg7/ATG7 in murine and human cell lines) prevents starvation‐induced ACBP/DBI release (Bravo‐San Pedro, Sica, Martins, Pol, et al., 2019). Although there is consensus that core proteins involved in the autophagic machinery are required for ACBP/DBI release, there may be species‐specific pathways involving for instance the Golgi‐associated protein GRASP in fungal but not in mammalian species (Duran et al., 2010; Kinseth et al., 2007). In mice, a partial autophagy defect due to the knockout of *Atg4b* is sufficient to prevent the starvation‐induced release of ACBP/DBI from cells contained in the heart, kidney, liver, and muscle (Bravo‐San Pedro, Sica, Martins, Pol, et al., 2019). Similarly, drugs that interfere with the initiation of autophagy (such as dimethyl‐α‐ketoglutarate) (Baracco et al., 2019) or the fusion of autophagosomes with lysosomes (such as 3‐hydroxychloroquine) (Boya et al., 2005) can be injected into mice to inhibit the starvation‐induced release of ACBP/DBI into the circulation (Bravo‐San Pedro, Sica, Martins, Pol, et al., 2019). Mechanistically, it appears that, in human cells, activation of the pro‐autophagic enzyme AMP‐activated protein kinase (AMPK) results in the phosphorylation of ACBP/DBI on serine 21, causing its dissociation from phosphatidylethanolamine (Udupa et al., 2022), a lipid species that is associated with autophagosomes and stimulates autophagy (Rockenfeller et al., 2015). However, whether the AMPK‐induced desorption of ACBP/DBI from lipids is necessary and sufficient for the cellular release of ACBP/DBI remains an open question.

In conclusion, there is ample evidence that in fungi and animal cells ACBP/DBI is usually present in the cytosol of cells, yet can be secreted in an autophagy‐dependent manner to reach the extracellular compartment.

## 3 PHYLOGENY OF ACBP/DBI RECEPTORS

Protein secretion by unicellular fungi (or any organism) would be a mere waste of energy if the secreted protein had no extracellular function. Accordingly, in *S*. *cerevisiae*, extracellular ACBP acts on the surface receptor Ste3p, a G protein‐coupled 7 transmembrane receptor (GPCR), which is also the receptor for the pheromone a‐factor, thus stimulating a MAPK kinase cascade (Versele et al., 2001).

In *D*. *discoideum*, the ACBP/DBI ortholog AcbA is proteolytically processed to the peptide signal spore differentiation factor 2 (SDF‐2), which acts on a membrane‐associated histidine kinase, DhkA, on pre‐spore cells to inhibit its activity and to stimulate the intracellular accumulation of cyclic AMP (cAMP) and the consequent activation of protein kinase A (Anjard & Loomis, 2005).

In mice, extracellular ACBP interacts with a specific isoform of the heteropentameric GABA_A_ receptor, which is a gated chloride channel that is composed by 1 α‐chain, 2 β‐chains, and 2 γ chains (each with several isoforms). For ACBP binding, the GABA_A_ receptor must contain the γ2 isoform (GABRG2). A point mutation (F77I) in GABRG2 abrogates ACBP binding (Anagnostopoulos et al., 2022), as well as the effects of ACBP on neurogenesis (Dumitru et al., 2017). This mutation also abolishes the binding and hypnotic effects of zolpidem (Cope et al., 2005), in line with the fact that ACBP/DBI and benzodiazepines act on the same receptor, which has been dubbed as “central benzodiazepine receptor.” However, this nomenclature does not reflect the fact that GABA_A_ receptors including the γ2 isoform are expressed in various peripheral tissues (outside of the central nervous system, CNS) including colon (Saeed et al., 2012; Yan et al., 2020), liver, white adipose tissue (Anagnostopoulos et al., 2022), and T lymphocytes (Giannone et al., 2004).

In mice and rats, ACBP and its peptide fragment ODN also act on a pertussis toxin‐inhibitable ODN‐GPCR, and specific central nervous (anxiogenic and anorexigenic) effects of ACBP injected into the brain (intrathecally or stereotactically into the hypothalamus) can be blocked by ODN‐GPCR inhibition (Bouyakdan et al., 2019; Guillebaud et al., 2017). Thus, ODN induces pertussis toxin‐inhibitable Ca^2+^ fluxes in cultured rat astrocytes (Lamacz et al., 1996). As a caveat, ODN‐GPCR thus far is a hypothetical, purely pharmacologically defined entity and has not yet been identified in genetic terms.

Finally, in mammalian cells, intracellular ACBP/DBI interacts with translocator protein (TSPO), which was initially designated as “peripheral benzodiazepine receptor.” TSPO is an evolutionarily ancient protein with 5 transmembrane domains, exposed to the surface of mitochondria and plays roles in steroidogenesis and metabolic regulation (Bonsack & Sukumari‐Ramesh, 2018), as well as in neuroinflammation (Corica et al., 2023). However, it has not been shown that this ACBP/DBI‐TSPO interaction would stimulate specific signal transduction pathways.

**FIGURE 2:**
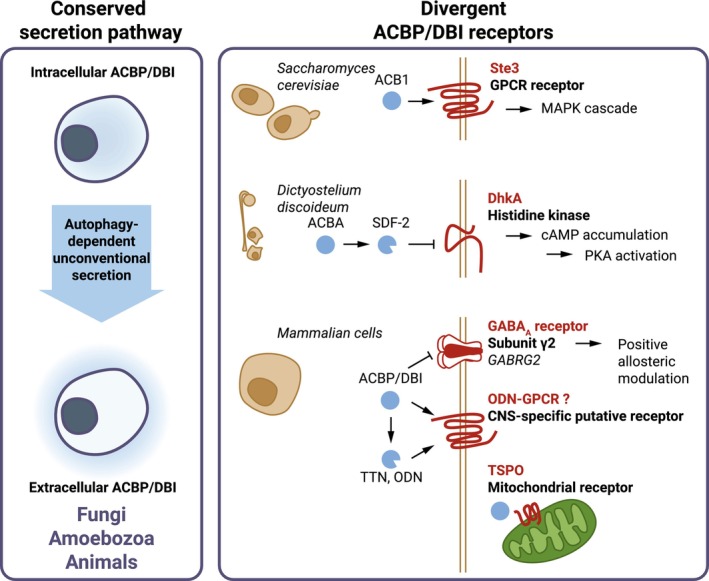
ACBP/DBI autophagy‐dependent secretion is conserved across phylae and targets diverse transmembrane receptors. Release of the intracellular ACBP/DBI upon autophagy induction has been demonstrated in fungal, amoebozoan, and animal species. Once in the extracellular compartment, a variety of transmembrane receptors induce different downstream molecular effects upon binding of ACBP/DBI (or its orthologs). These receptors include the GPCR receptor Ste3 in *Saccharomyces cerevisiae*, the receptor histidine kinase DhkA in *D*. *discoideum*, the γ2 subunit of the extracellular GABAA receptor (GABRG2), the mitochondrial membrane receptor TSPO and a putative central nervous system‐specific GPCR receptor (ODN‐GPCR). ACBP, acyl CoA binding protein; DBI, diazepam binding inhibitor; GABA_A_R, gamma‐amino butyric acid receptor type A; GABRG2, GABA_A_R subunit γ2; GPCR, G‐protein coupled receptor; ODN, octadecaneuropeptide; TSPO, translocator protein; TTN, triakontatetraneuropeptide.

In sum, although ACBP/DBI protein and its autophagy‐dependent release are highly conserved throughout phylogeny, extracellular ACBP/DBI acts on a variety of different receptors, namely a receptor histidine kinase (in *D*. *discoideum*), metabotropic GPCRs (in *S*. *cerevisiae* and perhaps in rodents), and ionotropic GABA_A_ receptors (in mice and human cells), indicating that the signaling pathways activated by ACBP/DBI have diverged during evolution across distinct phyla (Figure 2). This observation is not unique in thus far that ligand‐receptor coevolution is often influenced by pleiotropy exhibited by polypeptides (Jiang et al., 2014). The question rather arises whether the functional output of such signaling pathways still demonstrates some commonality. Moreover, the question arises whether other ACBP/DBI receptors remain to be discovered.

## 4 ACBP/DBI ORTHOLOGS IN FUNGI, PLANTS, AND NON‐MAMMALIAN ANIMALS

**FIGURE 3:**
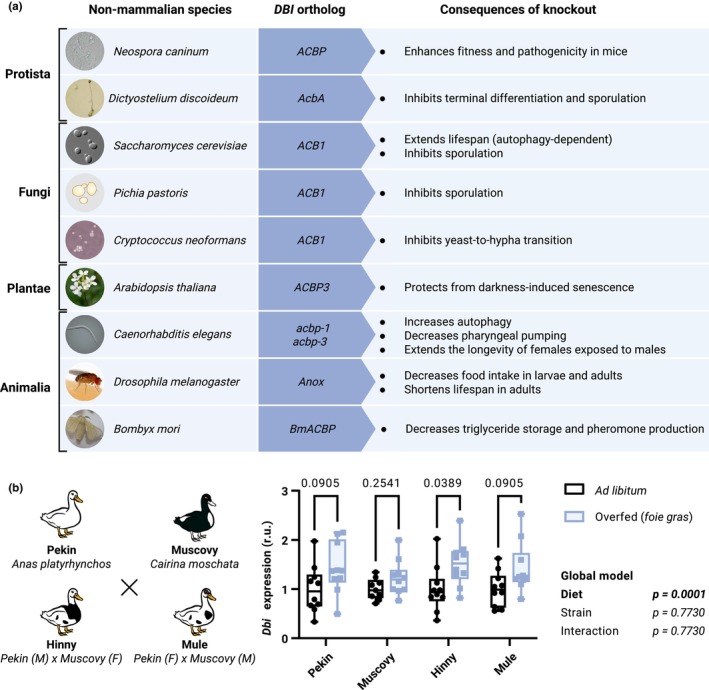
*DBI* orthologs are functionally linked to nutrient stress responses and aging across phylae. Phenotypic characterization of organisms deficient for *DBI* orthologs reveal their impact on age‐related pathways such as nutrient sensing, response to stress, and senescence in protista, fungi, plantae, and animalia species (a). RNA sequencing data from the livers of ducks from the two distinct species (Pekin and Muscovy ducks) and their hybrids (Hinny and Mule ducks) reveal that, overfeeding a high‐carbohydrate diet employed for *foie gras* production, *Dbi* expression is increased compared to ad libitum conditions (b). Data are extracted from (Herault et al., 2019). Statistical significance was tested by two‐way ANOVA, and multiple comparison was corrected with Holm–Šídák's test in GraphPad Prism (v9.5.1 for Windows, GraphPad Software www.graphpad.com).

In the yeast *S*. *cerevisiae*, knockout of *Acbp1* extends lifespan in chronological aging experiments that were conducted to systematically screen for genes affecting longevity (Fabrizio et al., 2010). Heat‐induced cell death was also diminished by *Acbp1* deletion, suggesting that this molecular manipulation increases the fitness of yeast cells in laboratory conditions (Montegut, Joseph, Chen, Abdellatif, Ruckenstuhl, Motino, et al., 2023). Similarly, the knockout of *ACBP* in *Neospora caninum* enhanced the fitness and pathogenicity of this parasite in mice (Zhou et al., 2020), again suggesting that, in specific circumstances, the absence of ACBP/DBI orthologs can confer an advantage for organismal fitness (Figure 3a).

Interestingly, *Acbp1* deletion (or that of its receptor *Ste3*) inhibited nitrogen starvation‐induced sporulation in *S*. *cerevisiae*, suggesting that ACBP/DBI orthologs may be important for the adaptation to nutritional stress (Charmpilas et al., 2020). Similarly, in *Pichia pastoris*, deletion of *Acb1* inhibits sporulation (Manjithaya et al., 2010), and in *Cryptococcus neoformans*, *Acb1* knockout inhibits yeast‐to‐hypha transition, which constitutes another adaptation to nutrient stress. In the same vein, in *D*. *discoideum*, knockout of *AcbA* abolishes terminal differentiation of the fruit body and subsequent sporulation (Anjard & Loomis, 2005). Altogether, these finding suggest that the improved fitness of ACBP/DBI‐deficient fungal or protist species detected in nutrient‐rich conditions (see above) manifests at the price of the failure to adopt a long‐term strategy (sporulation, differentiation) to nutrient‐scarce conditions.

In the best‐studied plant species, *A*. *thaliana*, transgenic overexpression of the sole extracellular ACBP/DBI ortholog, *ACBP3*, enhances leaf sclerosis and senescence, while its knockout attenuates leaf senescence (Xiao et al., 2010; Xiao & Chye, 2010). Similarly, in *Brassica napus*, an *ACBP3* ortholog, *BnACBP1‐like*, induces leaf chlorosis and senescence upon its transgenic overexpression (Ling et al., 2018), thus supporting an evolutionarily conserved pro‐aging function of ACBP/DBI orthologs. Of note, transgenic overexpression of autologous *ACBP3* (or xenogeneic expression of that of the *ACBP3* ortholog from rice, *Oryza sativa*) in *A*. *thaliana* confers protection against bacterial and fungal infection (Panthapulakkal Narayanan et al., 2019).

ACBP/DBI orthologs have been studied in several non‐mammalian animal species. In the nematode *Caenorhabditis* elegans, knockdown of *acbp1* (also called *maa‐1*) enhances longevity of adult worms (Shamalnasab et al., 2017). Moreover, the knockdown of *acbp1* alone or the simultaneous knockdown of several ACBP/DBI orthologs (*acbp1*, *acbp3*, *acbp4*, *acbp6*) reduced pharyngeal pumping (Charmpilas et al., 2020). Interestingly, the expression of *acbp3* is enhanced in female *C*. *elegans* upon exposure to sperm, whereas knockdown of *acb3* in the intestine is sufficient to reduce the accelerated demise of females induced by male nematodes (Booth et al., 2022).

In the fruit fly *Drosophila melanogaster*, knockout of an ACBP/DBI ortholog denoted *Anox* causes reduced insulin receptor gene expression in and severely attenuated food intake larval and adult flies, compromising organismal survival. Conversely, *Anox* overexpression increased food intake in larvae (Ryuda et al., 2011). In another insect, the silk worm *Bombyx mori*, knockout of *BmACBP* results in reduced triacylglyceride content of lipid droplets and decreased body fat (Ohnishi et al., 2006). This is phenocopied by treatment with pyridostatin, a drug that stabilizes G‐quadruplex (G4) structures and reduces *BmACBP* expression (Xiang et al., 2022).

Gene expression data from birds also suggest a role for ACBP/DBI in body weight control. Thus, in the wild duck (*Anas platyrhynchos*), reduced body weight gain under a probiotic fermented food diet is associated with reduced *DBI* mRNA expression in the liver (Gu et al., 2022). Overfeeding different duck species (*A*. *platyrhynchos*, *Cairina moschata* and their hybrids) with a carbohydrate‐rich diet causes the upregulation of *DBI* mRNA in the liver (Herault et al., 2019). Hence, the development of *foie gras* is linked to the overexpression of *DBI* (Figure 3b). Of note, in the Northern wheatear (*Oenanthe oenanthe*), reducing daylight exposure, which mimics pro‐migratory signals, enhances food intake and weight gain with steatosis, correlating with an increase in hepatic *DBI* mRNA (Frias‐Soler et al., 2022). Thus, both forced and voluntary overfeeding triggers increased *DBI* expression in the liver.

In conclusion, data obtained in fungi, plants, nematodes, and birds suggest the involvement of ACBP/DBI orthologs in the response to nutrient stress (Figure 3). Thus, ACBP/DBI orthologs stimulate starvation‐induced differentiation (exemplified by spore formation in unicellular fungi and *D*. *discoideum*), feeding behavior (in *C*. *elegans* and insects), and weight gain (in silk worms and birds). Another overarching observation concerns a potential pro‐aging effect of ACBP/DBI orthologs, at least in yeast, plants, and nematodes.

## 5 FUNCTIONAL EXPLORATION OF ACBP/DBI IN NON‐HUMAN MAMMALS

**FIGURE 4:**
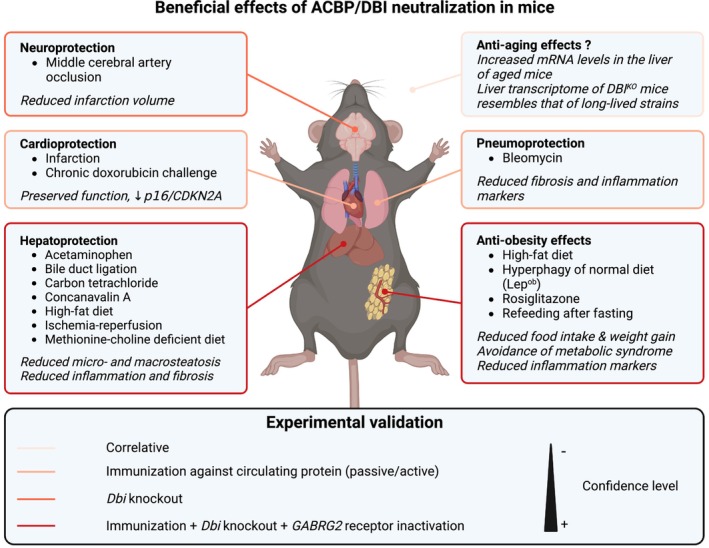
ACBP/DBI neutralization has systemic and organ‐specific protective effects in mice. Evidence for protective effects of ACBP/DBI neutralization has been demonstrated in murine models of obesity, organ‐specific damage (liver, heart, lung, brain), and aging, with various levels of confidence ranging from correlative clues to knockout of ACBP/DBI or knockin mutations of its downstream receptor GABRG2. Created with BioRender.com.

Most of the results dealing with the pathophysiological exploration of ACBP/DBI in mammals have been obtained in mice (*Mus musculus*) (Figure 4). Homozygous knockout of *Dbi* in W4/129S6 embryonic stem cells causes pre‐implantation embryonic lethality (Landrock et al., 2010). In C57BL/6 mice, *Dbi* has been knocked out at the constitutive level, meaning that the genetic deficiency comes into action during early development. When female and male C57BL/6 *Dbi* ^+/−^ mice are crossed, *Dbi* ^−/−^ mice are born at the expected Mendelian frequency. However, such C57BL/6 *Dbi* ^−/−^ mice exhibit a postnatal defect of skin epithelial integrity, leading to increased transepidermal water loss and death around weaning (Neess et al., 2011, 2013). This effect likely reflects a cell‐autonomous contribution of ACBP/DBI to lipid metabolism in the skin because knockout of *Dbi* in keratinocytes alone recapitulates this phenotype (Neess et al., 2013). In spite of this limitation, the liver transcriptome of *Dbi* ^−/−^ mice (determined at 3 weeks) exhibits a positive correlation with that of long‐lived mouse strains and a negative correlation with that of short‐lived strains (Fuentealba et al., 2021), supporting a pro‐aging effect of ACBP/DBI. Moreover, in animals conducted in young mice, *Dbi* KO confers resistance to stroke induced by middle cerebral artery occlusion (Lamtahri et al., 2021) but enhanced susceptibility to the parkinsonian neurotoxin 1‐methyl‐4‐phenyl‐1,2,3,6‐tetrahydropyridine (MPTP) (Bahdoudi et al., 2018).

In contrast to the constitutive knockout (KO), tamoxifen inducible KO of *Dbi* in young adult (6‐week‐old) C57/BL/6 mice does not compromise the survival of the animals, hence facilitating the long‐term exploration of ACBP/DBI effects (Bravo‐San Pedro, Sica, Martins, Pol, et al., 2019). Similarly, constitutive mutation of the ACBP/DBI receptor (*Gabrg2* ^F77I^), if present on both alleles (*Gabrg2* ^F77I/F77I^), can abolish signaling through ACBP/DBI without compromising animal survival (Anagnostopoulos et al., 2022). This contrasts with the postnatal lethality of the *Gabrg2* knockout (*Gabrg2* ^−/−^; Gunther et al., 1995), likely reflecting the requirement of this subunit for the maintenance of GABA_A_ receptors at mature synapses (Schweizer et al., 2003). Using these methods of ACBP/DBI inhibition, it has been shown that both inducible *Dbi* KO in adult mice and constitutive *Gabrg2* ^F77I/F77I^ mutation indistinguishably reduce weight gain and glucose intolerance with high‐fat diet, as well as liver damage by methionine‐choline‐deficient diet (Anagnostopoulos et al., 2022; Bravo‐San Pedro, Sica, Martins, Pol, et al., 2019; Joseph et al., 2021; Motino, Lambertucci, Anagnostopoulos, Li, Nah, et al., 2022). Moreover, *Dbi* KO protects adult mice from weight gain induced by long‐term rosiglitazone administration (which, in wild type mice, causes the upregulation of *Dbi* mRNA through an effect on the transcription factor PPARγ) (Anagnostopoulos et al., 2022) and enhanced weight loss upon switching from high‐fat to normal diet (Bravo‐San Pedro, Sica, Martins, Pol, et al., 2019). The resistance against high‐fat diet‐induced obesity observed after the inducible whole‐body *Dbi* KO could be recapitulated by a cell type‐specific constitutive knockout of *Dbi* in white adipose tissue (Joseph et al., 2021), supporting a role for ACBP/DBI in local adipogenesis, perhaps by favoring adipocyte differentiation, which is stimulated by ACBP/DBI in a cell‐autonomous fashion (Mandrup et al., 1998). Conditional knockout and overexpression of ACBP/DBI (or ODN) in neuronal stem cells reduce and enhance adult neurogenesis, respectively, and these effects of ACBP/DBI overexpression are lost in *Gabrg2* ^F77I/F77I^ mice (Dumitru et al., 2017). These results plead in favor of a positive role of ACBP/DBI in neurogenesis.

Additional methods for inhibiting ACBP/DBI consist of the induction of auto‐antibodies by repeated vaccination with a keyhole limpet hemocyanin (KLH)‐conjugated ACBP/DBI protein plus adjuvant (Bravo‐San Pedro, Sica, Martins, Pol, et al., 2019; Montegut, Chen, et al., 2022) or injections of neutralizing anti‐ACBP/DBI monoclonal antibody (mAb) (Bravo‐San Pedro, Sica, Martins, Pol, et al., 2019). Using these methods of active and passive immunization, it could be shown that the neutralization of ACBP/DBI reduced food intake upon fasting and refeeding (Bravo‐San Pedro, Sica, Martins, Pol, et al., 2019), attenuated weight gain after rosiglitazone treatment (Anagnostopoulos et al., 2022), and enhanced weight loss upon switching from high‐fat to normal diet (Bravo‐San Pedro, Sica, Martins, Pol, et al., 2019). ACBP/DBI neutralization also reduced weight gain in leptin‐deficient *Ob*/*Ob* mice (Bravo‐San Pedro, Sica, Martins, Pol, et al., 2019). Moreover, ACBP/DBI inhibition attenuated liver damage by acetaminophen, carbon tetrachloride, concanavalin A, high‐fat diet, methionine‐choline‐deficient diet, ischemia reperfusion and bile duct ligation (Motino, Lambertucci, Anagnostopoulos, Li, Nah, et al., 2022), reduced heart damage by infarction (Motino, Lambertucci, Anagnostopoulos, Li, Nah, et al., 2022) or chronic doxorubicin administration (Montegut, Joseph, Chen, Abdellatif, Ruckenstuhl, Motino, et al., 2023), and attenuated lung fibrosis induced by bleomycin (Motino, Lambertucci, Anagnostopoulos, Li, Nah, et al., 2022). All these effects were coupled to a reduction of inflammation, as indicated by histological examination or reduced expression of pro‐inflammatory genes.

Recombinant ACBP/DBI has been injected intravenously (i.v.) to stimulate food intake, and this effect is lost in *Gabrg2* ^F77I/F77I^ mice (Joseph et al., 2020). In contrast, the Y29F and K33A mutations of ACBP/DBI (which abolish its interaction with acyl CoA) did not compromise the appetite‐stimulatory effects of i.v. injected ACBP/DBI protein (Joseph et al., 2020). Thus, the extracellular action of ACBP can be separated from its acyl CoA binding ability. The food intake‐stimulating (orexigenic) and obesogenic effects could be recapitulated by hydrodynamic injection of ACBP/DBI‐encoding vectors that cause transgenic overexpression of the protein in the liver commensurate with an increase in circulating ACBP/DBI protein levels (Bravo‐San Pedro, Sica, Martins, Pol, et al., 2019). In sharp contrast, injection of recombinant ACBP/DBI protein or its neuropeptides (or that of ACBP7 and its neuropeptide) (Lanfray et al., 2016) into the brain via different routes (intrathecal, intracerebroventricular, or intra‐hypothalamic) had anxiogenic and anorexigenic effects (De Mateos‐Verchere et al., 1998, 2001) that were inhibited by the metabotropic ODN‐GPCR receptor antagonist cyclo1–8[DLeu^5^] octapeptide (Arg‐Pro‐Gly‐Leu‐DLeu‐Asp‐Leu‐Lys; CDLOP) (Bouyakdan et al., 2019; do Rego et al., 2007; Guillebaud et al., 2017), the peripheral (extra‐CNS) and cerebral effects of ACBP/DBI on food intake appear to be mediated by distinct receptors and diametrically opposed in their functional outcome. These findings indirectly argue against the possibility that i.v. ACBP/DBI injected would directly act on the CNS, in line with the reported incapacity of ACBP/DBI to cross the blood–brain barrier (Barmack et al., 2004). Rather, ACBP/DBI detected in the plasma may stimulate appetite in an indirect fashion, likely due metabolic effects resulting in reduced circulating glucose and free fatty acids (Bravo‐San Pedro, Sica, Martins, Pol, et al., 2019). That said, in specific circumstances a centralized pool of ACBP/DBI (that is lost upon conditional knockout of *Dbi* in GRAP^+^ astrocytes) does stimulate appetite, for instance in the context of refeeding after a fasting period (Bouyakdan et al., 2022), suggesting that the debate whether peripherally injected ACBP/DBI (or its neuropeptides) might reach specific brain centers to stimulate feeding behavior is yet to be resolved.

**FIGURE 5:**
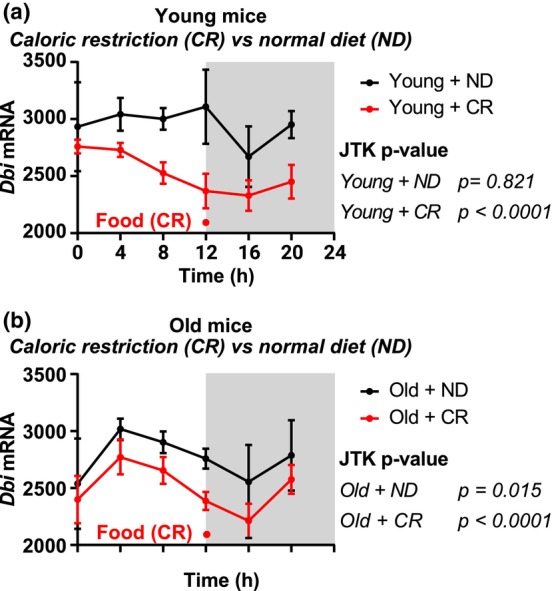
Daily oscillations of ACBP/DBI are increased by calorie‐ and time‐restricted feeding in mice. ACBP/DBI transcripts counts were extracted from RNA sequencing data of livers from young (<1 year old) and old (1.5–2 years old) mice, collected every 4 h for 24 h (data from (Sato et al., 2017). Mild to inexistant oscillatory patterns are visible in young (a) and old (b) mice fed ad libitum with chow diet, while clear daily oscillation occurs when mice are given a single dose of calorie‐restricted food at the time of light extinction. Cycling variable identification was tested with the Jonckheere‐Terpstra‐Kendall algorithm, implemented in R (https://www.R‐project.org/) with the the *MetaCycle* package (Wu et al., 2016). CR, caloric restriction; JTK, Jonckheere‐Terpstra‐Kendall; ND, normal diet.

ACBP/DBI mRNA and protein levels in the liver increase with aging, high‐fat diet‐induced obesity of normal mice or obesity of leptin‐deficient *Ob*/*Ob* mice (Anagnostopoulos et al., 2022; Bravo‐San Pedro, Sica, Martins, Pol, et al., 2019; Wang et al., 2021). Interestingly, ACBP/DBI mRNA levels in the murine suprachiasmatic nucleus and liver are subjected to circadian regulation (Hughes et al., 2009; Pembroke et al., 2015), commensurate with circadian oscillations of GABRG2 mRNA in the paraventricular hypothalamus (Kim et al., 2020). Daily oscillations of ACBP/DBI mRNA in the liver were modest in young and old mice under a normal (moderately obesogenic diet), and this oscillation (which occurred at generally lower levels) was increased in conditions of caloric restriction (Figure 5), in line with the general rule that circadian regulation improves in health‐improving conditions (Lopez‐Otin & Kroemer, 2021). Of note, high‐fat diet‐induced obesity correlates with increased myocardial ACBP/DBI mRNA levels, and this latter effect is not reversed by semaglutide treatment (Pan et al., 2022). Finally, ACBP/DBI mRNA levels increase with obesity in arterial and venous endothelial cells from both visceral and subcutaneous adipose tissue from mice (Bondareva et al., 2022).

The association of ACBP/DBI with obesity is also found in other mammalian species than mice. Thus, in the rat (*Rattus norvegicus*), high‐fat diet increases Dbi protein levels in heart, kidney, and liver (Bhuiyan et al., 1995). Dbi protein augments in epididymal adipose tissue of Wistar rats fed a high‐caloric Western diet (Berti et al., 2012), in muscles from diabetic Zucker rats (that overconsume a normal diet because they lack leptin receptors) (Franch et al., 2002), and in livers from wild type rats treated with the cholestase inducer α‐naphthylisothiocyanate (Yao et al., 2021). In pigs (*Sus domesticus*), ACBP/DBI mRNA levels in muscle correlate with fat content (Yang, Zhang, et al., 2022). A relationship between ACBP/DBI and inflammation is suggested by reports indicating an increase in plasma protein levels in rats subjected to cecal ligation and puncture (CLP) to induce peritonitis (Clavier et al., 2014). Moreover, ACBP/DBI mRNA levels increase in the left ventricle of hypertrophic hearts from the spontaneously hypertensive Okamoto strain as compared to aged‐matched normal rats (Dwyer et al., 2008).

Altogether, the aforementioned results suggest that, in mice, ACBP/DBI neutralization has marked organ‐protective effects against ischemia (in the brain, heart, and liver) and other types of physical damage (bile duct ligation), as well as against the toxic effects of a variety of compounds (acetaminophen, bleomycin, carbon tetrachloride, concanavalin A but not the Parkinsonian toxin MPTP). In addition, ACBP/DBI appears to contribute to the pathophysiology of obesity and may be involved in inflammation (Figure 4).

## 6 DISEASE‐ASSOCIATED ALTERATIONS OF ACBP/DBI IN HUMANS

**FIGURE 6:**
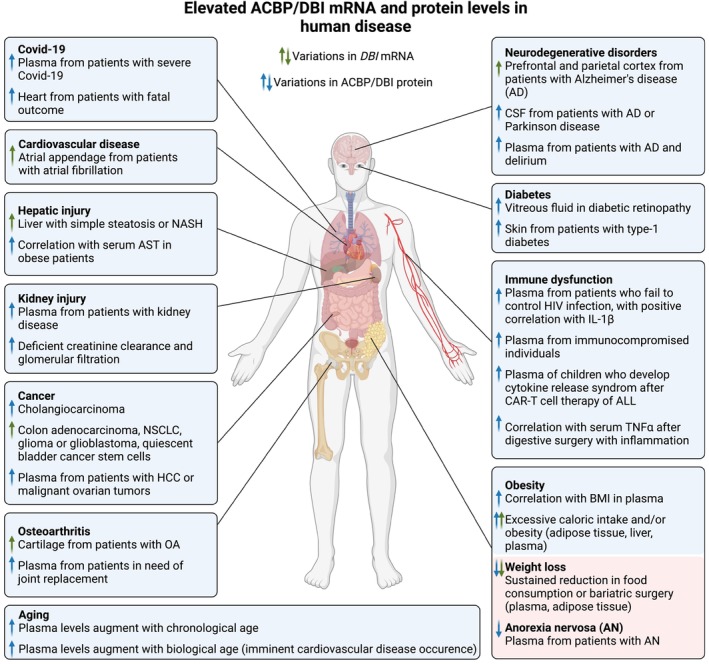
Protein and mRNA levels of ACBP/DBI in various human diseases. ACBP/DBI levels extracted from public large‐scale transcriptomics and proteomics datasets or measured specifically in human samples are elevated in a variety of human diseases. For references, see main text. Created with BioRender.com.

There is ample evidence that ACBP/DBI levels change in human diseases (Figure 6). Before we discuss the level of ACBP/DBI protein in peripheral blood (plasma or serum), we will examine disease‐associated alterations of ACBP/DBI mRNA or protein in specific organs.

ACBP/DBI mRNA levels were found upregulated in adipose tissue biopsies upon doubling caloric intake by fast food (Franck et al., 2011). Conversely, ACBP/DBI mRNA was reduced after feeding a very low‐calorie diet, but increased after refeeding (Franck et al., 2011). Similarly, in periumbilical fat, ACBP/DBI mRNA was enhanced in obese patients and reduced after a 6 week‐long hypocaloric diet or bariatric surgery (Bravo‐San Pedro, Sica, Martins, Pol, et al., 2019). Local ACBP/DBI mRNA levels also correlated with subcutaneous fat volume in patients infected by human immunodeficiency virus (HIV) (Gabriel et al., 2021), further supporting a likely obesogenic action of ACBP/DBI. In liver biopsies, ACBP/DBI mRNA levels are higher in both simple steatosis and nonalcoholic steatohepatitis (NASH) compared to healthy controls (Arendt et al., 2015). With respect to healthy controls, ACBP/DBI mRNA is upregulated in peripheral blood mononuclear cells from patients with chronic fatigue syndrome (Saiki et al., 2008), in atrial appendage tissue from patients with persistent atrial fibrillation with or without heart failure (Zeemering et al., 2022), in the cartilage from patients with osteoarthritis (Shorter et al., 2022), and in the prefrontal and parietal cortex from Alzheimer disease patients (Luchetti et al., 2011; Mills et al., 2013). Accordingly, ACBP/DBI protein levels measured by enzyme‐linked immunosorbent assay (ELISA) are increased in the cerebrospinal fluid from patients with dementia including Alzheimer and Parkinson diseases (Ferrarese et al., 1990; Ferrero et al., 1988).

ACBP/DBI has been implicated in the pathogenesis of diabetes. In patients with type‐1 diabetes, ACBP/DBI levels determined by mass spectrometry are elevated in skin biopsies, and this elevation is reversed by curative kidney and pancreas transplantation (Folli et al., 2010). Enhanced levels of ELISA‐detectable ACBP/DBI are evident in vitreous samples correlating with age, in particular in proliferative diabetic retinopathy compared to non‐diabetic controls (Gao et al., 2022). In addition, a non‐confirmed genetic association study suggests that two single nucleotide polymorphisms of *DBI* that likely reduce the expression of ACBP/DBI are linked to a reduced risk of developing type‐2 diabetes in two German study populations (Fisher et al., 2007).

Several studies suggest the implication in ACBP/DBI in malignant disease. In breast cancer patients, high expression of ACBP/DBI mRNA levels has a negative prognostic impact on progression‐free and overall survival (Jacob et al., 2023). Mass‐spectrometric proteomics indicates that ACBP/DBI is highly abundant in colon adenocarcinoma compared to normal tissue (Atak et al., 2018). Immunohistochemical detection of ACBP/DBI also reveals high expression in cholangiocarcinoma (Cavalloni et al., 2021). ACBP/DBI mRNA levels are upregulated in non‐small cell lung cancers (NSCLC), correlating with poor overall survival, cancer stage, and patient smoking history. In vitro, knockdown of ACBP/DBI reduced NSCLC cell proliferation, suggesting a cell‐autonomous effect (Harris et al., 2014). Similarly, ACBP/DBI is highly expressed in glioma and glioblastoma, and its knockdown reduced proliferation and induced senescence in vitro or after transplantation into immunodeficient mice (Duman et al., 2019). ACBP/DBI is also strongly expressed in quiescent bladder cancer stem cells, correlating with high histological grade (Yao et al., 2022). Whether this tumor‐associated overexpression of ACBP/DBI only involves tumor cell‐intrinsic effects or may also lead to reprogramming of the tumor microenvironment by secreted ACBP/DBI has not been elucidated.

Most studies dealing with ACBP/DBI in human disease report changes in the plasma concentration that usually have been measured by ELISA, radioimmunoassay (RIA), proximal extension assay (Olink), or aptamer‐based proteomic assay (SomaScan). As a caveat, however, quantitations obtained by two commercially provided proteomic platforms, Olink and SomaScan, do not correlate consistently (average correlation *r* = 0.46) (Rooney et al., 2023), meaning that results based on these platforms may require additional validation steps.

ELISA based quantification of plasma ACBP/DBI indicates a strong positive correlation with body mass index (BMI). Accordingly, ACBP/DBI is reduced in anorexia nervosa (Bravo‐San Pedro, Sica, Martins, Pol, et al., 2019; Conti et al., 2013) but increased in obese individual and then declines after weight reduction due to bariatric surgery (Bravo‐San Pedro, Sica, Martins, Pol, et al., 2019; Joseph et al., 2020, 2021). In addition, plasma ACBP/DBI concentrations correlate with chronological age in adults. This correlation is statistically independent from that with BMI (Joseph et al., 2021; Montegut, Joseph, Chen, Abdellatif, Ruckenstuhl, Motino, et al., 2023). ACBP/DBI also correlates with biomarkers of pre‐diabetes (fasting glucose and insulin) (Bravo‐San Pedro, Sica, Martins, Pol, et al., 2019; Montegut, Joseph, Chen, Abdellatif, Ruckenstuhl, Motino, et al., 2023), liver damage (serum aspartate transaminase) in obese patients (Bravo‐San Pedro, Sica, Martins, Pol, et al., 2019), reduced renal function (glomerular infiltration, high creatine levels) (Joseph et al., 2021), as well as with several cardiovascular disease (CVD) risk factors (high total cholesterol, triglycerides, systolic blood pressure, reduced HDL cholesterol) (Joseph et al., 2020, 2021; Montegut, Joseph, Chen, Abdellatif, Ruckenstuhl, Motino, et al., 2023). ELISA‐quantified ACBP/DBI is elevated in patients with Alzheimer disease and delirium, in particular in the hyperkinetic form (Conti et al., 2021). Moreover, high plasma ACBP/DBI are associated with failure to control HIV‐1 infection, correlating with circulating interleukin‐1β (Isnard et al., 2022). Similarly, RIA‐quantifiable plasma ACBP/DBI levels correlate with plasma tumor necrosis factor‐α levels after digestive surgery with inflammation (Clavier et al., 2014) and are elevated in patients with hepatocellular carcinoma and cirrhosis compared to individuals with cirrhosis alone (Venturini et al., 1998).

Both Somascan‐ and Olink‐based proteomics convergently indicate that plasma ACBP/DBI is increased in the context of coronavirus disease‐19 (COVID‐19). This applies to early acute phase of COVID‐19 versus healthy controls. The ACBP/DBI increase is associated with disease severity in COVID‐19 patients, as well as higher risk of clinical failure and longer hospital stay. It is also enhanced in patients with post‐acute sequelae of COVID‐19 compared to individuals with uncomplicated recovery. Clinically, ACBP/DBI is significantly enhanced in patients with kidney disease and tends to be elevated in immunocompromised individuals (Bauer et al., 2023; Filbin et al., 2021; Paranjpe et al., 2022; Talla et al., 2021). Accordingly, ACBP/DBI protein is enhanced in the heart from patients with fatal COVID‐19, as determined by mass spectrometry (Nie et al., 2021). Moreover, Olink‐based measurements of plasma ACBP/DBI indicate an elevation in patients (*n* = 1181) that undergo joint replacement due to osteoarthritis within 2 years (±2 years) of plasma collection as compared to other samples from the UK biobank (*n* = 49,754) (Styrkarsdottir et al., 2023), an increase in the plasma of patients with malignant ovarian tumors compared with benign tumor‐bearers (Gyllensten et al., 2022), as well as an elevation of plasma levels in children who develop severe cytokine release syndrome after CAR T therapy against acute B lymphocyte leukemia compared to children who do not manifest cytokine release syndrome (Diorio et al., 2022).

In summary, in humans, ACBP/DBI is elevated in old age, individuals with cardiometabolic risk factors, as well as patients with a variety of pathologies including diabetes, obesity, uncontrolled infection or inflammatory, neurodegenerative and malignant diseases (Figure 6). The only human disease in which ACBP/DBI is reduced is anorexia nervosa. The association of ACBP/DBI with chronological age and with multiple age‐related diseases suggests that the ACBP/DBI plasma concentration is a proxy of biological age. Accordingly, plasma levels of ACBP/DBI in still healthy individuals that will develop cardiovascular events within 3–9 years of follow‐up are higher than in age‐ and BMI‐matched controls that remain disease free in this timeframe (Montegut, Joseph, Chen, Abdellatif, Ruckenstuhl, Motino, et al., 2023). Thus, high levels ACBP/DBI may constitute a biomarker of biological aging (leading to early manifestation of CVD) beyond its correlation with chronological age.

## 7 MECHANISTIC INSIGHTS REGARDING ACBP/DBI INHIBITION

As discussed above, ACBP/DBI is a phylogenetically old protein that is released from nutrient‐stressed cells through an evolutionarily conserved autophagy‐dependent pathway but then acts on a diverse array of receptors that are fundamentally distinct in yeast, slime molds, and mammals. Nonetheless, ACBP/DBI appears to play a conserved pro‐aging effect in the sense that its genetic or pharmacological inhibition confers anti‐aging effects (in yeast, plants, and nematodes), combats metabolic syndrome, and protects numerous organs (including brain, heart, liver, and lung) against pathogenic cell loss, inflammation, and fibrosis (in mice). Epidemiological evidence derived from human samples also suggest an implication of ACBP/DBI in aging and age‐related diseases. The question then arises whether the broad anti‐aging and pro‐health effects of ACBP/DBI neutralization involve a common mechanistic denominator.

Based on the current state of the literature, it appears possible that the enhancement of autophagic flux resulting from ACBP/DBI inhibition may contribute to its beneficial effects. This speculation is based on the established role of autophagy in the maintenance of health and the avoidance of age‐associated disease including NASH, CVD, cancer, and neurodegeneration (Abdellatif et al., 2018; Aman et al., 2021; Klionsky et al., 2021; Levine & Kroemer, 2019; Lopez‐Otin et al., 2023; Lopez‐Otin & Kroemer, 2021) Moreover, there are numerous pieces of evidence in favor of the conjecture that ACBP/DBI neutralization acts through the induction of autophagy.

For example, in *S*. *cerevisiae*, genetic inhibition of autophagy by knockout of essential autophagy‐related genes such as *Atg5* or *Atg7* abolished the longevity‐extending effect of the *Acbp1* deletion (Montegut, Joseph, Chen, Abdellatif, Ruckenstuhl, Motino, et al., 2023). Similarly, in *A*. *thaliana*, the leaf senescence‐inducing effect of *ACBP3* has been linked to disabled autophagy (Xiao et al., 2010; Xiao & Chye, 2010). In *C*. *elegans*, reduced caloric intake and consequent autophagy induction in the gut account for the lifespan‐extending effect of the *eat‐2* mutation (Gelino et al., 2016), suggesting that a similar mode of action might explain the longevity conferred by *maa‐1*/*acbp‐1* inhibition, which indeed reduces pharyngeal pumping and stimulates autophagy (Charmpilas et al., 2020).

While elevations of plasma ACBP/DBI (by i.v. injection of the recombinant protein or hepatocyte‐specific transgenesis) enhance food intake in mice (which inhibits autophagy), neutralization of ACBP/DBI by mAbs reduces food intake (which induces autophagy), suggesting that the peripheral (extra‐CNS) pool of ACBP/DBI inhibits autophagy through neuroendocrine circuitries (Bravo‐San Pedro, Sica, Martins, Pol, et al., 2019). However, autophagy induction by ACBP/DBI inhibition is also found in vitro, in cultures of human and murine cells, indicating the existence of autocrine or paracrine circuitries through which ACBP/DBI suppresses autophagy (Bravo‐San Pedro, Sica, Martins, Anagnostopoulos, et al., 2019; Bravo‐San Pedro, Sica, Martins, Pol, et al., 2019).

In mice, knockout‐ or antibody‐mediated inhibition of ACBP/DBI enhances autophagic flux in all investigated organs including heart, liver, and muscle (Bravo‐San Pedro, Sica, Martins, Pol, et al., 2019; Montegut, Joseph, Chen, Abdellatif, Ruckenstuhl, Motino, et al., 2023; Motino, Lambertucci, Anagnostopoulos, Li, Nah, et al., 2022). Pharmacological inhibition of autophagy by repeated injections of 3‐hydroxychloroquine abolishes the hepatoprotective effects of antibody‐mediated ACBP/DBI neutralization against acetaminophen, carbon tetrachloride, concanavalin A, methionine‐choline‐deficient diet, ischemia reperfusion, and bile duct ligation (Motino, Lambertucci, Anagnostopoulos, Li, Martins, & Kroemer, 2023; Motino, Lambertucci, Anagnostopoulos, Li, Nah, et al., 2022). Similarly, knockout of essential autophagy genes annihilates cardioprotection and hepatoprotection by an anti‐ACBP/DBI mAb. Thus, cardiomyocyte‐specific knockout of *Atg7* abolishes the cardioprotective effect of anti‐ACBP/DBI mAb in a model of cardiac ischemia/reperfusion. The body‐wide knockout of *Atg4b* precludes hepatoprotection by anti‐ACBP/DBI mAb against methionine/choline‐deficient diet (Motino, Lambertucci, Anagnostopoulos, Li, Nah, et al., 2022). In this NASH model, inhibition of *Atg4b* also abolishes the favorable gene transcription program induced by the neutralizing anti‐ACBP/DBI mAb. This concerns the anti‐ACBP/DBI‐stimulated downregulation of pro‐inflammatory and pro‐fibrotic genes as well as the upregulation of antioxidant enzymes and the ß‐oxidation‐inducing carnitine palmitoyl transferase‐1, which are detectable in wild type but not in *Atg4b* ^−/−^ mice (Motino, Lambertucci, Anagnostopoulos, Li, Nah, et al., 2022). Thus, at least in NASH, all the favorable changes induced by neutralization of extracellular ACBP/DBI appear to occur downstream of the ignition of autophagy.

In anthracycline‐induced accelerated cardiac aging, ACBP/DBI neutralization does not only improve the function of the heart but also reduces the frequency of senescent cells expressing cyclin‐dependent kinase inhibitor 2A (CDKN2A, best known as p16) (Montegut, Joseph, Chen, Abdellatif, Ruckenstuhl, Motino, et al., 2023), which has previously been causatively involved in DOX‐induced cardiac failure (Demaria et al., 2017). Whether this anti‐senescence effect is secondary to autophagy induction has not been determined, although autophagy reportedly has anti‐senescence effects in other contexts (Aman et al., 2021; Klionsky et al., 2021).

In conclusion, it appears that ACBP/DBI acts as a tonic autophagy inhibitor in several distinct phyla in spite of the divergent evolution of ACBP/DBI receptors. However, at this point there is little mechanistic information on the proximal signals that link ACBP/DBI receptors to autophagy inhibition a part from the fact that chloride channels can regulate autophagy in mammalian cells (as this has been described for cystic fibrosis conductance receptor, CFTR) (Luciani et al., 2012; Zhang et al., 2019).

## 8 CONCLUDING REMARKS

Based on its phylogenetic ancestry, ACBP/DBI may well be (one of) the oldest polypeptide hormone(s) that developed during evolution. Usually an intracellular protein, ACBP/DBI can be released through an unconventional, autophagy‐dependent mechanism into the extracellular space. There, ACBP/DBI interacts with a diverse array of cell surface‐exposed receptors that are not conserved among phyla. Notwithstanding the divergent evolution of ACBP/DBI receptors, it appears that extracellular ACBP/DBI inhibits autophagy in several distinct species through autocrine, paracrine, and neuroendocrine circuitries. Thus, ACBP/DBI communicates the activation of intracellular stress pathways (autophagy) to other cells, thereby participating to the maintenance of systemic homeostasis (Galluzzi et al., 2018).

ACBP/DBI mediates the adaptation of model organisms to fluctuating food sources through distinct mechanisms that range from sporulation (in fungi and slime molds) to enhanced nutrient intake and lipid storage (in nematodes, insects, birds, and mice), hence switching from lipo‐catabolism to lipo‐anabolism. This metabolic switch is mediated by a surge in extracellular ACBP/DBI due to its autophagy‐dependent release from multiple different cell types (in conditions of starvation) or due to its transcriptional upregulation (in conditions of overnutrition) and involves the ACBP/DBI‐mediated suppression of autophagy. Indeed, in conditions of severe nutrient stress it may be advantageous to limit autophagy, which, constitutes an instance of self‐consumption (Marino et al., 2014) and, as shown for cases of extreme anorexia, may even cause the death (rather than the stress adaptation) of cells via “autosis” (Fernandez et al., 2020; Kheloufi et al., 2015). However, it remains to be determined whether extra supply of ACBP/DBI would have a positive effect on anorexia, a condition in which ACBP/DBI levels are subnormal. Moreover, two pathologies that reportedly mediated by autosis (such as ischemia/reperfusion damage of the myocardium and doxorubicin‐induced cardiomyopathy) (Nah et al., 2022) are attenuated rather than aggravated by ACBP/DBI neutralization, shedding doubts on the conjecture that low ACBP/DBI levels would favor autosis.

Perhaps as a result of antagonistic pleiotropy, in fungi, slime molds, plants, nematodes, and mice, ACBP/DBI has pro‐aging effects that appear secondary to the inhibition of autophagy, which undoubtedly constitutes one of the most important endogenous anti‐aging mechanisms (Levine & Kroemer, 2019; Lopez‐Otin et al., 2016, 2023). In mice, ACBP/DBI contributes to the pathogenesis of a variety of age‐related diseases ranging from obesity to NASH, liver or lung fibrosis, myocardium infarction, and anthracycline‐induced cardiac aging. In humans, elevations of ACBP/DBI mRNA or protein affect various tissues as well as the blood stream in the context of aging, diabetes, obesity, uncontrolled infection, or cardiovascular, inflammatory, neurodegenerative, and malignant diseases. Direct experimentation in mouse models and literature‐based speculation suggest that many if not most of the pro‐aging effects of ACBP/DBI are mediated by autophagy inhibition.

In conclusion, ACBP/DBI can be considered as an aging‐ and disease‐relevant “autophagy checkpoint.” ACBP/DBI‐specific antibodies neutralize extracellular ACBP/DBI and hence act as “autophagy checkpoint inhibitors” (ACI), thereby stimulating autophagy. We have coined this denomination by analogy to the “immune checkpoint inhibitors” (ICI) targeting the immunosuppressive interaction between PD‐1 and PD‐L1. In the same way as ICI have become the backbone of most oncological treatments in a broad range of distinct indications, ACI might turn out to be useful for prevention and treatment of a wide spectrum of pathological states including aging, cardiovascular, infectious, inflammatory, malignant, and metabolic diseases that are associated with insufficient autophagy.

## AUTHOR CONTRIBUTIONS

This paper was written by LM and GK with input by all co‐authors. Figures were drawn by LM. All co‐authors have reviewed the paper and concur with its submission.

## CONFLICT OF INTEREST STATEMENT

GK has been holding research contracts with Daiichi Sankyo, Eleor, Kaleido, Lytix Pharma, PharmaMar, Osasuna Therapeutics, Samsara Therapeutics, Sanofi, Tollys, and Vascage. GK is on the Board of Directors of the Bristol Myers Squibb Foundation France. GK is a scientific cofounder of everImmune, Osasuna Therapeutics, Samsara Therapeutics, and Therafast Bio. GK is in the scientific advisory boards of Hevolution, Institut Servier, and Longevity Vision Funds. GK is the inventor of patents covering therapeutic targeting of aging, cancer, cystic fibrosis, and metabolic disorders. GK's wife, Laurence Zitvogel, has held research contracts with Glaxo Smyth Kline, Incyte, Lytix, Kaleido, Innovate Pharma, Daiichi Sankyo, Pilege, Merus, Transgene, 9 m, Tusk, and Roche, was on the on the Board of Directors of Transgene, is a cofounder of everImmune, and holds patents covering the treatment of cancer and the therapeutic manipulation of the microbiota. GK's brother, Romano Kroemer, was an employee of Sanofi and now consults for Boehringer‐Ingelheim. F.M. is a scientific cofounder of Samsara Therapeutics, and has equity interests in and is advisor of The Longevity Labs (TLL). LM, MA, OM, IM, and GK are listed as co‐inventors on ACBP/DBI‐relevant patents. The funders had no role in the design of the study, in the writing of the manuscript, or in the decision to publish the results.

## Data Availability

Data sharing is not applicable to this article as no new data were created or analyzed in this study.
